# Glutamic acid assisted hydrolysis strategy for preparing prebiotic xylooligosaccharides

**DOI:** 10.3389/fnut.2022.1030685

**Published:** 2022-10-17

**Authors:** Rong Huang, Rui Zhang, Shuangquan Yao, Mengyuan Si, Ruowen Xia, Xin Zhou, Xingli Fan, Kankan Jiang

**Affiliations:** ^1^School of Basic Medical Sciences and Forensic Medicine, Hangzhou Medical College, Hangzhou, China; ^2^Jiangsu Co-innovation Center of Efficient Processing and Utilization of Forest Resources, College of Chemical Engineering, Nanjing Forestry University, Nanjing, China; ^3^Guangxi Key Laboratory of Clean Pulp & Papermaking and Pollution Control, School of Light Industry and Food Engineering, Guangxi University, Nanning, China

**Keywords:** prebiotics, xylooligosaccharides, glutamic acid, xylan, hydrolysis

## Abstract

Since the immune-boosting properties as well as the benefit of promoting the growth of gut bacteria, xylooligosaccharides as prebiotics have attracted considerable interest as functional feed additives around the world. A growing number of studies suggest that acidic hydrolysis is the most cost-effective method for treating xylan materials to prepare xylooligosaccharides, and organic acids were proved to be more preferable. Therefore, in this study, glutamic acid, as an edible and nutritive organic acid, was employed as a catalyst for hydrolyzing xylan materials to prepare xylooligosaccharides. Further, xylooligosaccharide yields were optimized using the response surface methodology with central composite designs. Through the response surface methodology, 28.2 g/L xylooligosaccharides with the desirable degree of polymerization (2–4) at a yield of 40.5 % could be achieved using 4.5% glutamic acid at 163°C for 41 min. Overall, the application of glutamic acid as a catalyst could be a potentially cost-effective method for producing xylooligosaccharides.

## Introduction

In parallel with the emergence of health consciousness in the consumer market, natural and healthier food resources are being embraced, such as prebiotic products and preventive medicines (1). Prebiotics are a class of substrates that can be selectively utilized by beneficial microorganisms in the host, conferring health benefits (2). The common prebiotics include lactitol, lactosucrose, fructooligosaccharides (FOS), mannanoligosaccharides (MOS), xylooligosaccharides (XOS), galactooligosaccharides (GOS), isomaltooligosaccharides (IMO), and milk-oligosaccharides (3, 4). Among these oligosaccharides prebiotics, XOS, as a class of oligomers that comprise 2–10 xylose monomeric units, are defined as non-digestible food or feed additives and are proved to feature in the proliferation of beneficial microorganisms (5, 6). XOS with dietary supplementation of 100 mg/kg can effectively improve the proportion of probiotics (*Lactobacillus* and *Bifidobacterium*) and enhance the amount of short-chain fatty acids; these gut intestinal probiotics and the biologically generated short-chain fatty acids contribute significantly to boosting intestinal development and regulating immunity function (5, 7). As feed additives, a small addition of XOS can effectively improve the growth performance of animals and reduce mortality in animal breeding (8, 9).

As a promising prebiotic product, the XOS can be sold in China at market prices ranging between 25 $ and 50 $ per Kg. The relatively high market prices of XOS products have driven more and more researchers to perform the study of advanced and simple methods for preparing XOS (10). XOS has been reported to naturally appear in fruits, vegetables, and bamboo shoots; however, the extraction of XOS was very difficult as its concentration in these materials is too low (11). The XOS products can be produced from lignocellulosic materials rich in xylan by enzymatic or acidic hydrolysis methods (5, 12). The method of enzymatic hydrolysis can realize high purity XOS production, however, the main drawbacks of the method of enzymatic hydrolysis are the loss of enzymes activities and they are unrecyclable, resulting in a higher cost; meanwhile, the enzymatic hydrolysis process requires a relatively prolonged time, casing a lower output (13, 14).

Relatively speaking, acidic hydrolysis methods, by rapidly breaking the glycosidic linkages in the case of the specific hydrogen ionic environment, are more suitable or preferred for industrial-scale XOS production (15–17). Based on previous literature, both mineral acids and organic acids are capable of assisting in hydrolyzing xylan-rich materials into XOS products (18, 19). However, the main drawback of mineral acids hydrolysis is the generation of more byproducts, such as xylose and furfural, reducing the XOS production; moreover, the use of mineral acids can result in the large formation of inorganic effluent (20). Different from mineral acids, organic acids are generally weak acids and have desirable features of less equipment corrosiveness and fewer byproducts, which are more preferable for producing XOS with a higher yield (17, 21). Theoretically, organic acids, such as gluconic acid, maleic acid, citric acid and xylonic acid, could be directly co-prepared with XOS and applied as feed additives (17, 19, 22–24).

In this study, glutamic acid (GluA), as an edible and nutritive additive, is also able to release hydronium ions (H^+^) to randomly cleave xylan into XOS and xylose. GluA, as one kind of amino acid, which occurs naturally in proteinaceous foods such as meats, seafood, stews, soups, sauces, is nontoxic and harmless for animals or humans (25, 26). The XOS and GluA can be co-prepared as a mixture and applied in feed or food additives. Therefore, GluA was introduced as the acid catalyst to assist the hydrolysis of xylan, which was extracted from sugarcane bagasse, into XOS products (27). As XOS production was the primary objective of this work, response surface methodology (RSM) was used to optimize the conditions of GluA concentration, reaction temperature, and hydrolysis period (28, 29).

## Materials and methods

### Materials

The xylan powder was extracted from sugarcane bagasse, which was collected from Guangxi Province, China. The dried sugarcane bagasse mainly comprises 43.5% glucan, 27.9% xylan, 23.8% lignin, and 2.1% ash content. Generally, 1,000 g sugarcane bagasse with sizes of 40–120 mesh was mixed with 10 L 10% (w/v) NaOH in a 15-L stainless steel rotary pot, and the alkaline treatment was conducted at 120°C for 1 h; the mixture was filtered after alkaline treatment and the pH was adjusted to 5 by adding H_2_SO_4_ (27). The xylan was then precipitated with an equal volume of 95% ethanol (v/v). The precipitated xylan was centrifuged at 6,000 rpm for 10 min and washed with ice-cold ethanol for three times. Finally, the precipitated xylan was freeze-dried for further study. The purity of the freeze-dried xylan sample was 69.5%, which was analyzed according to the protocol of National Renewable Energy Laboratory (30). Besides, the Klason lignin content and acid soluble lignin in the xylan sample were respectively 18.5% and 3.8%.

### Glutamic acid assisted hydrolysis of xylan

Five grams of xylan powder from sugarcane bagasse was added in a 100 mL screw-top pressure-resistant steel tube reactor, which contained 50 mL 1–7% GluA solution with the solid-liquid ratio of 1:10. Then the tube was placed in an oil bath at a preset temperature (130–180°C) and heated for 30–70 min. The reactions were stopped at the scheduled arrival time; the mixture was centrifuged at 6,000 rpm for 10 min. Then the supernatant containing XOS was harvested for subsequent analysis.

### Design of single-factor experiments and response surface method

Single-factor screening experiments were performed to assess the effect of reaction temperature, GluA concentration, and hydrolysis period for xylan hydrolysis. Firstly, in the case of the fixed 5% GluA concentration and 50 min hydrolysis period time, the effect of the reaction temperature was tested at 130–180°C. Then, in the case of the fixed 160°C reaction temperature and 50 min hydrolysis period, the effect of the GluA concentration was evaluated with 1–9%. Lastly, in the case of the fixed 160°C reaction temperature and 5% GluA concentration, the effect of the hydrolysis period was investigated with the time of 30–70 min.

Following the single-factor experimental results, a suitable point was selected and subjected to Design-Expert ^®^ (Version 11.0) for designing response surface experiments. This experiment was conducted with reaction temperature (150, 160, and 170°C), GluA concentration (3, 5, and 7% w/w), and hydrolysis time (30, 50, and 70 min) as the independent variables. A 3^3^ factorial design was elaborated and listed in Table 1, which showed the detailed experiments conducted in triplicate. One-way analysis of variance (ANOVA) and Duncan's multiple range test (*p* < 0.05) were used for analyzing the statistical significance. The relationship between the response (XOS yield) and independent variables [reaction temperature (*x*_1_), GluA concentration (*x*_2_), and hydrolysis period (*x*_3_)] was calculated through the following quadratic polynomial equation.


$$\begin{array}{l} Y\;=\;a_{0}+\sum a_{i}x_{i}+\sum a_{ii}x_{i}^{2}+\sum a_{ij}x_{i}x_{j} \end{array}$$

**Table 1:** Different combination of independent variables of software design and experimental results.

**Variables**			**Responses**	**Byproducts**	
***x*_1_: Reaction temperature (^o^C)**	***x*_2_: GluA^a^ concentration (% w/w)**	***x*_3_: Hydrolysis period (min)**	**Y: XOS^b^ yields (%)**	**Xylose(%)**	**Furfural (%)**
150	3	50	16.54	3.92	0.17
150	5	30	6.34	1.42	0.00
150	7	50	24.83	9.40	1.08
150	5	70	32.15	18.63	1.14
160	3	30	17.69	4.21	0.00
160	7	70	17.20	48.58	3.71
160	5	50	40.30	26.17	1.81
160	3	70	31.49	31.71	1.22
160	7	30	29.47	10.86	1.49
170	5	30	34.92	18.38	3.43
170	5	70	12.28	60.81	5.00
170	3	50	36.80	51.33	3.97
170	7	50	17.24	28.21	1.05

a GluA, glutamic acid.

b XOS, xylooligosaccharides.

In this equation, Y represents the XOS yield as a response, a_0_ is a constant term, *x*_i_ and *x*_j_ are independent variables, and a_i_, a_ii_, and a_ij_ are coefficients of linear, quadratic, and interaction parameters, respectively.

### Analytical methods

Furfural and xylose were simultaneously measured through the high performance liquid chromatograph (Agilent 1260) equipped with the column of Aminex Bio-Rad HPX-87H (Bio-Rad Laboratories). XOS, including xylobiose (X2), xylotriose (X3), xylotetraose (X4), xylopentaose (X5), and xylohexaose (X6), were determined through high performance anion exchange chromatography (HPAEC) (Thermo ICS-5000) equipped with the column of CarboPac^TM^ PA200 (Thermo) (31). The following are the equations used to calculate the yields of xylose, furfural, and XOS.

$$\begin{array}{l} Furfural\;yield\;\left(\%\right)=\frac{Furfural\;content\;in\;hydrolyates\;\left(g\right)}{Initial\;xylan\;content\;\left(g\right)}\\ \times 100\%\\ Xylose\;yield\;\left(\%\right)=\frac{Xylose\;content\;in\;hydrolyates\;\left(g\right)}{Initial\;xylan\;content\;\left(g\right)}\times 100\%\\ XOS\;yield\;\left(\%\right)\\ =\frac{X2+X3+X4+X5+X6\;content\;in\;hydrolyates\;\left(g\right)}{Initial\;xylan\;content\;\left(g\right)}\\ \times 100\% \end{array}$$

## Results and discussion

### Influence of single factors on the acidic hydrolysis process of xylan

**Figure 1:**
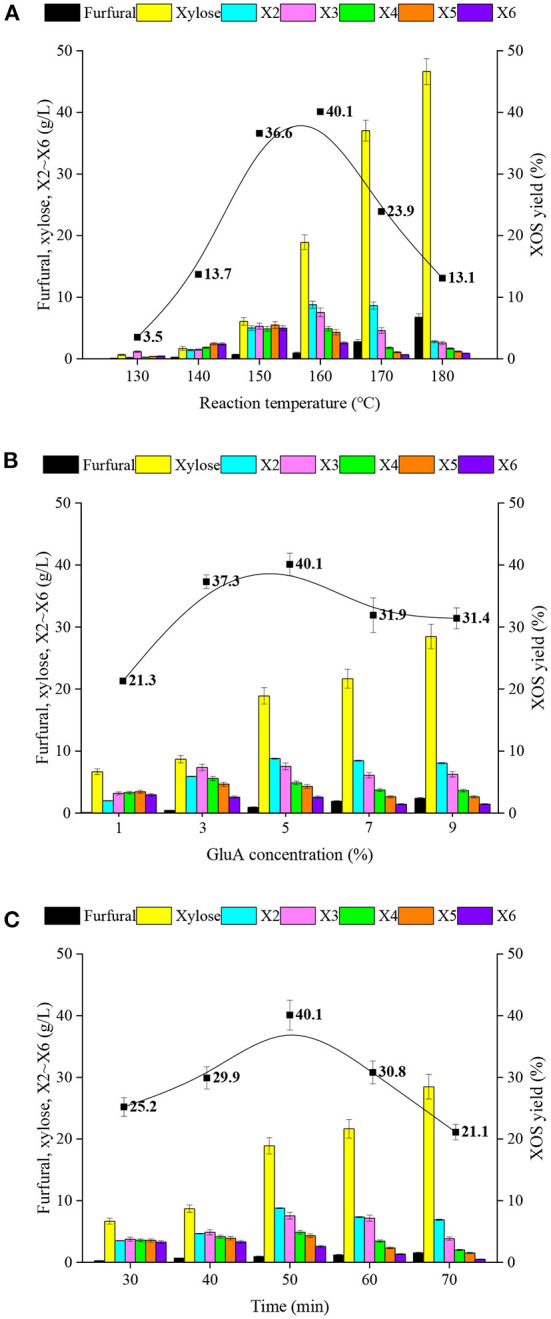
The effects of **(A)** reaction temperature, **(B)** GluA concentration and **(C)** hydrolysis period for the products, including the content of furfural, xylose, X2-X6, and the yields of XOS in GluA-hydrolysate of xylan. X2, xylobiose; X3, xylotriose; X4, xylotetraose; X5, xylopentaose; X6, xylohexaose; XOS, xylooligosaccharides.

XOS production has been reported to be significantly influenced by process parameters, such as reaction temperature, acid concentration, and hydrolysis period (17, 32). Therefore, single-factor experiments were used to examine the effects of these variables on XOS production and xylan degradation in this study, and the results were described in Figure 1. It could be observed that XOS yields increased progressively as the reaction temperature increased, and reached a peak (the content and yield of XOS were 27.9 g/L and 40.1%) at 160°C, before declining as the reaction temperature increased further. In addition, when the reaction temperature was <150°C or higher than 170°C, XOS yields were relatively low.

The release of H^+^ from GluA during the process of acidic hydrolysis can contribute to breaking glycosidic linkages of xylan. Xylan was first hydrolyzed into saccharides with relatively high DP; subsequently, these saccharides were further degraded into oligomers (33, 34). As the reaction continues, the oligomers would also be hydrolyzed into xylose and furfural (17). Higher temperature provides much higher activation energy for dehydration since it releases H^+^ from organic acid more readily, which results in more saccharides being degraded to XOS and xylose. The evidence also showed that the hydrolysis reaction rates could be accelerated with increased reaction temperatures, namely, the excessively high reaction temperature would cause a lower content of XOS with a low degree of polymerization (DP), the proportion of X2 gradually increased and the rate of accumulation of small molecular compounds such as xylose and furfural also increased synchronously. Figure 1A showed that the yields of xylose and furfural were increased greatly while the reaction temperature was over 170°C. In the case of 180°C, the yield of xylose from xylan hydrolysis was nearly 50%. These results suggested that the reaction temperature strength should be controlled at 150–170°C.

Figure 1B showed the effects of GluA concentration on the degradation of xylan. Similarly, as the GluA concentration was raised, XOS production increased gradually, attained a maximum value with GluA concentration of 5%, and then declined as the GluA concentration rose above 7%. In comparison with reaction temperature, the changes in GluA concentration did not show strong effects on the XOS yield. As shown in Figure 1B, the XOS yield gradually increased from 21.3 to 40.1% with the GluA concentration increasing from 1 to 5%. However, a further increase in GluA concentration resulted in a slight decline in the XOS yield. Approximately 31% XOS yield was obtained with 7–9% GluA. Apparently, under the same hydrolysis period and reaction temperature conditions, the higher GluA loading provides more H^+^, thereby accelerating the xylan hydrolysis and forming more byproducts (xylose and furfural).

Figure 1C displayed the effect of the hydrolysis period for XOS yield, which increased with the extended reaction time at a certain range (30–50 min). With the increased duration, the high DP oligomers, such as X5 and X6, would be further hydrolyzed into lower DP oligomers (X2 and X3). It could be observed that the amounts of X5 and X6 declined and the amounts of X2 and X3 continued to ascend with a longer retention period. In addition, the inordinately long duration also gave rise to the decomposition of XOS into xylose, even furfural, which caused the drop in the XOS yield. All results suggested that the realization of desirable DP distribution with relatively high XOS yield was dependent on all three key factors.

### Response surface method optimization for maximizing xylooligosaccharides yields

The results based on the single-factor experiments revealed that the maximum XOS yield was found to be at 160°C with 5% GluA for 50 min. In addition to elucidating the effect of individual variable and their interactions, RSM can provide an empirical model. To determine the optimal conditions, a three level-three factor (including 13 experimental runs) central composite design (CCD) was used for modeling the experimental process (Table 1) (35). In this study, three independent variables, GluA concentration (3–7%), reaction temperature (150–170^o^C), and hydrolysis period (30–70 min) were considered to discuss their effect on the yield of XOS (**Y**). A corresponding XOS yield (average value) under different conditions was determined by repeating the designed assays for three times. Herein, *x*_1_, *x*_2_, and *x*_3_ represent reaction temperature, GluA concentration, and hydrolysis period, respectively. After multiple regression analyses using Design-Expert software, the quadratic model was selected as the best fit to the Y. Following is the equation used to calculate the response value XOS:

$$\begin{array}{l} Y=35.42x_{1}+90.88x_{2}+111.83x_{3}-0.44x_{1}x_{2}-0.051x_{1}x_{3}\\ -0.16x_{2}x_{3}-0.095{x_{1}}^{2}-1.28{x_{2}}^{2}-0.028{x_{3}}^{2}-3335.88 \end{array}$$

**Figure 2:**
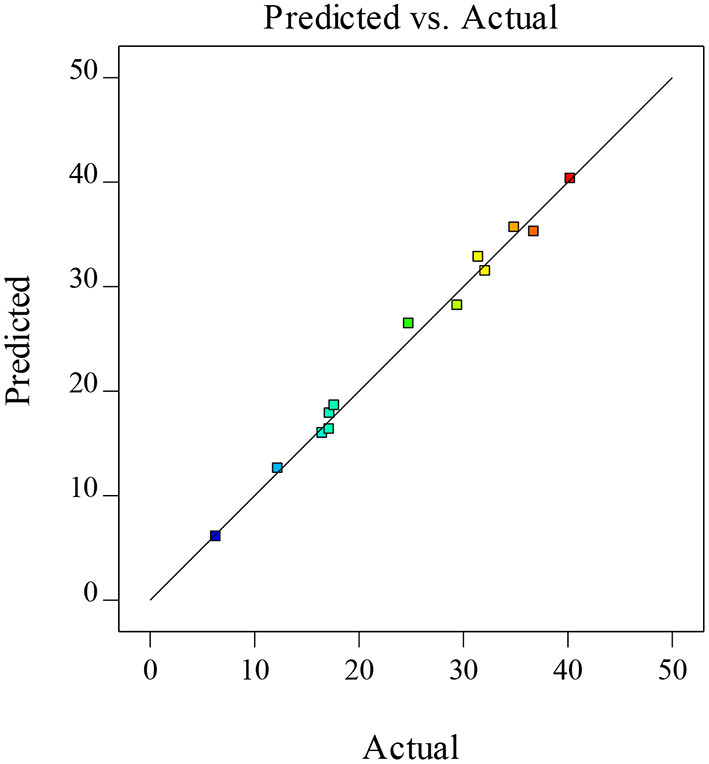
The plot for actual vs. predicted XOS yield.

A regression equation with a coefficient of determination R^2^ and an adjusted coefficient of determination R^2^ (Adj. R^2^) of more than 0.80 is considered to be a good fit. In this model, R^2^, and Adj. R^2^ were 0.9911 and 0.9644, showing an indication of the suitability of the fitted model for experimental results and predictions (36). In addition, Figure 2 represented the comparison between the predicted and the actual values of the XOS yields, which indicated that the difference between the actual and predicted values was small and the model was feasible.

The values from the ANOVA for the yields of XOS are depicted in Table 2. A coefficient's significance is always determined by its probability value (*P*-value), only variables that have the *P*-values lower than 0.05 for the regression model can be regarded as statistically significant, and lesser *P*-values signify a higher significance. The *P*-value and F-value were 0.0064 (<0.05) and 37.17, indicating that the selected model is significant. In this study, the *P*-values of *x*_1_, *x*_1_*x*_2_, *x*_1_*x*_3_, *x*_2_*x*_3_, *x*_1_^2^, *x*_2_^2^, *x*_3_^2^ were all <0.05, suggesting that they were significant model terms. In terms of the independent variable, only reaction temperature (*P*-value: 0.0320) was significant, whereas GluA concentration (*P*-value: 0.0916) and hydrolysis period (*P*-value: 0.4651) were insignificant, and the order of significance for three independent variables for the yield of XOS was: reaction temperature > GluA concentration > hydrolysis period.

**Table 2:** ANOVA for quadratic model.

**Source**	**Sum of squares**	**df**	**Mean square**	**F-value**	***P*-value**	
**Model**	1,324.41	9	147.16	37.17	0.0064	**Significant**
*x*_1_	57.12	1	57.12	14.43	0.0320	
*x*_2_	23.76	1	23.76	6.00	0.0917	
*x*_3_	2.76	1	2.76	0.6965	0.4652	
*x*_1_ *x*_2_	193.93	1	193.93	48.99	0.0060	
*x*_1_ *x*_3_	586.83	1	586.83	148.23	0.0012	
*x*_2_ *x*_3_	169.79	1	169.79	42.89	0.0072	
*x*_1_^2^	206.03	1	206.03	52.04	0.0055	
*x*_2_^2^	110.50	1	110.50	27.91	0.0132	
*x*_3_^2^	201.23	1	201.23	50.83	0.0057	
Residual	11.88	3	3.96			
Cor total	1,336.26	12				

**Figure 3:**
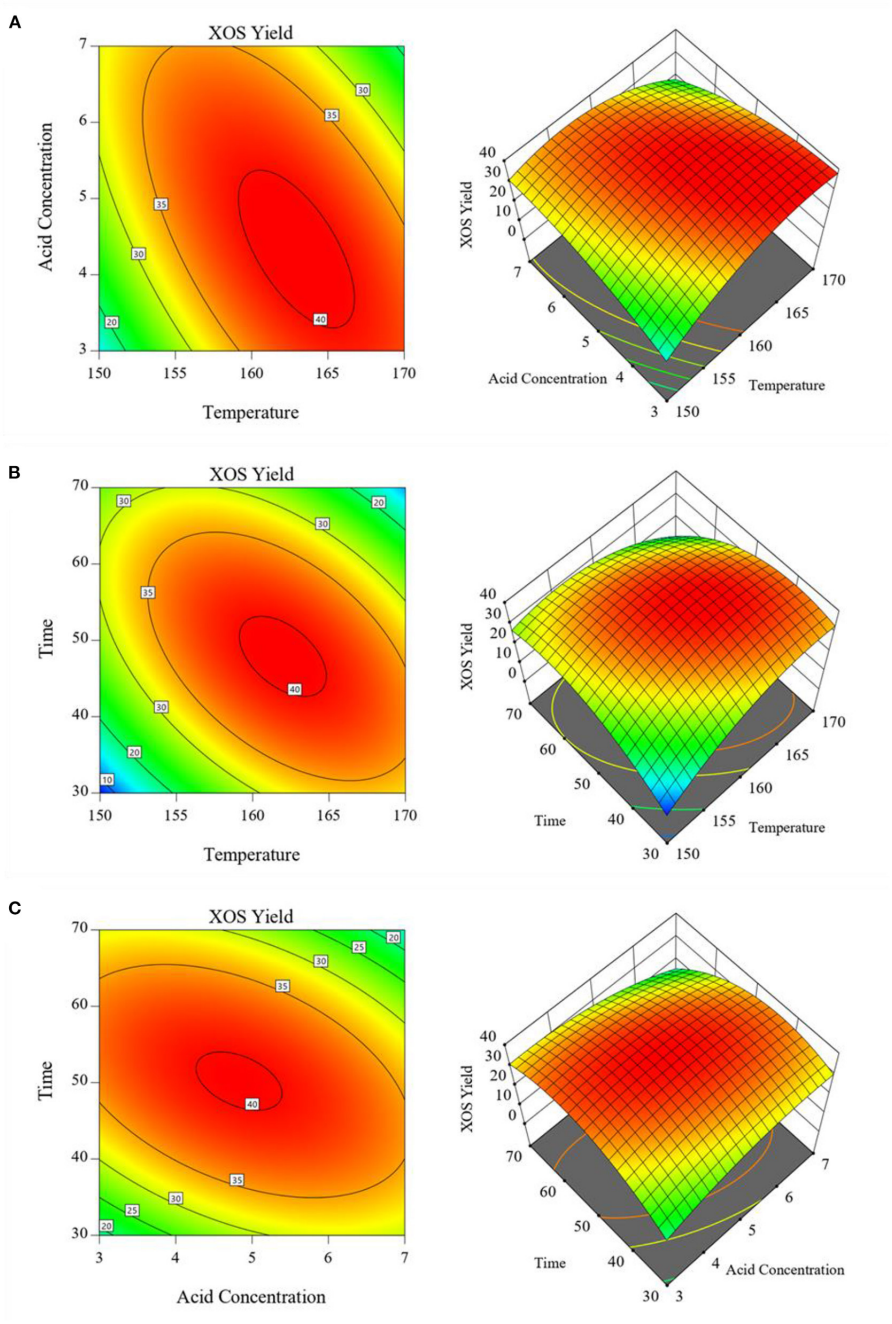
Response surface showing the effects of independent variables on XOS yields: **(A)** Reaction temperature and GluA concentration; **(B)** Reaction temperature and hydrolysis period; **(C)** GluA concentration and hydrolysis period. XOS, xylooligosaccharides.

In addition, reaction temperature × GluA concentration, reaction temperature × hydrolysis period, and GluA concentration × hydrolysis period were the three main groups of interactive factors. The response surface graphs of interactive effects between two independent variables for XOS yield as a function were depicted in Figure 3. As graphical representations of regression equations, two-dimensional (2D) contour plots and three-dimensional (3D) response surfaces can show the interactive effects of two factors, whereas the third one is fixed. A red zone in Figure 3 represents the ideal conditions for XOS production. It could be seen in Figure 3A, that when the reaction temperature ranged from 159 to 165°C and GluA concentration ranged from 3.7 to 5.2%, the maximal contour with the XOS yield over 40% could be achieved. At high reaction temperature or GluA concentration, increasing H^+^ concentration or temperature can aggravate the hydrolysis of xylan or oligomers, and even further cause the dehydration and acetylation of xylose. Therefore, too high values of both the two variables would lead to a decline in XOS yield and an increase in xylose yield. This phenomenon is in good agreement with the previous literature (23). Additionally, similar observations also could be observed in Figures 3B,C, which revealed the interaction effects of reaction temperature and hydrolysis period, as well as GluA concentration and hydrolysis period on XOS yield.

### Verification of the optimal results

**Figure 4:**
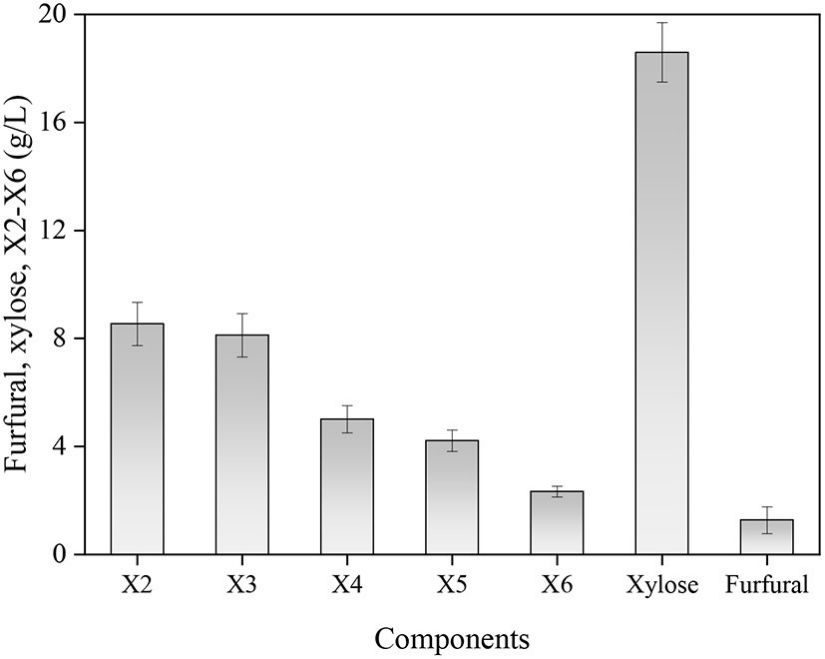
The distributions of the main components in hydrolysate under the optimum condition. X2, xylobiose; X3, xylotriose; X4, xylotetraose; X5, xylopentaose; X6, xylohexaose.

All results suggested that the changes occurring in the reaction temperature, GluA concentration and hydrolysis period would significantly invoke a change in obtaining XOS. To maximally obtain XOS, these three variables should be controlled in the ranges of 159–165°C, 3.7–5.2%, and 41–54 min, which achieved the XOS yield of over 40%. By applying the RSM, the optimum conditions for XOS yield were achieved: reaction temperature 162.608°C, GluA concentration 4.546%, and hydrolysis period 40.828 min. For sake of the convenient operation, the condition was selected as: reaction temperature 163°C, GluA concentration 4.5%, and hydrolysis period 41 min. For validation of the model, the experiment was conducted in triplicate under the optimum condition as predicted by the fitted model, and the distributions of the main components (furfural, xylose, X2–X6) in the hydrolysate were described in Figure 4.

Based on the results of the highest yield, the mean value was 40.5%, which corresponded to the predicted value (40.8%). The experimental result indicated that the actual result was consistent with the predicted result, confirming the accuracy of the formed quadratic polynomial equation. In previous studies, Zhang et al. (37) obtained a maximum XOS yield of 45.86% when using 20% acetic acid at 140°C for 20 min to hydrolyze waste xylan extracted from viscose fiber plants. A maximum 49.2% yield of XOS was achieved after applying 1.2% furoic acid to facilitate corncob xylan hydrolysis for 33 min at 167°C (38). Though the XOS yield in this study was lower than other organic acid hydrolysis, GluA catalysis for XOS production is still feasible as the advantage of low acid dosage and edibility. Moreover, the hydrolysate from xylan hydrolysis under the optimal condition contained 8.54 g/L X2, 8.12 g/L X3, 5.01 g/L X4, 4.21 g/L X5, and 2.31 g/L X6. Meanwhile, the xylose content was 18.67 g/L and the produced furfural was low (1.32 g/L). The distribution of XOS components showed that the main oligomers were X2–X4, which were considered to be the most effective compounds for prebiotics (39). The content of X2–X4 accounted for 84.2% of all XOS, whereas the proportion of X5 and X6 was relatively small, implying the GluA contributed to the acquisition of desirable DP of XOS.

## Conclusions

In this study, the edible glutamic acid as catalyst was initiatively used for preparing XOS *via* acidic hydrolysis of sugarcane bagasse-derived xylan. RSM approach following the single-factor experiments was employed to maximize the XOS yield and evaluate the effects of reaction temperature, acid concentration and hydrolysis period on the XOS yield. As a result, the maximum XOS yield was 40.5% using 4.5% glutamic acid at 163°C for 41 min. Under these conditions, desirable XOS degree of polymerization (2-4) accounting for 84.2% of all XOS was achieved, while the furfural was low. This study provides an insight into a promising and feasible method for large-scale production of prebiotic XOS.

## Data availability statement

The original contributions presented in the study are included in the article/supplementary material, further inquiries can be directed to the corresponding author/s.

## Author contributions

KJ and XF developed the idea and methodology for the study and helped in manuscript preparation and revision. RH and RZ performed the experiment and drafted the manuscript. SY, MS, RX, and XZ analyzed the results and interpreted them. All authors contributed to manuscript revision, read, and approved the submitted version.

## Funding

The study was supported by the Opening Project of Guangxi Key Laboratory of Clean Pulp & Papermaking and Pollution Control (2021KF25), Nanning, China, Innovation and Entrepreneurship Training Program for College Students of Jiangsu Province (202210298084Y), Basic Scientific Research Funds of Hangzhou Medical College (KYYB202002), and National Innovation and Entrepreneurship Training Program for College Students (202213023009).

## Conflict of interest

The authors declare that the research was conducted in the absence of any commercial or financial relationships that could be construed as a potential conflict of interest.

## Publisher's note

All claims expressed in this article are solely those of the authors and do not necessarily represent those of their affiliated organizations, or those of the publisher, the editors and the reviewers. Any product that may be evaluated in this article, or claim that may be made by its manufacturer, is not guaranteed or endorsed by the publisher.
